# Revealing the favorable dissociation pathway of type II kinase inhibitors *via* enhanced sampling simulations and two-end-state calculations

**DOI:** 10.1038/srep08457

**Published:** 2015-02-13

**Authors:** Huiyong Sun, Sheng Tian, Shunye Zhou, Youyong Li, Dan Li, Lei Xu, Mingyun Shen, Peichen Pan, Tingjun Hou

**Affiliations:** 1 Institute of Functional Nano and Soft Materials (FUNSOM), Soochow University, Suzhou, Jiangsu 215123, P. R. China; 2 College of Pharmaceutical Sciences, Zhejiang University, Hangzhou, Zhejiang 310058, P. R. China

## Abstract

How does a type II inhibitor bind to/unbind from a kinase target is still a confusing question because the small molecule occupies both the ATP pocket and the allosteric pocket of the kinase binding site. Here, by using enhanced sampling simulations (umbrella sampling, US) and two-end-state free energy calculations (MM/GSBA), we systemically studied the dissociation processes of two distinct small molecules escaping from the binding pocket of p38 MAP kinase through the allosteric channel and the ATP channel. The results show that the unbinding pathways along the allosteric channel have much lower PMF depths than those along the ATP channel, suggesting that the allosteric channel is more favorable for the dissociations of the two inhibitors and thereby supporting the general understanding that the largest channel of a target is usually the entry/exit pathway for the binding/dissociation of small molecules. Interestingly, the MM/GBSA approach yielded similar PMF profiles compared with those based on US, a much time consuming approach, indicating that for a general study, such as detecting the important transition state of a ligand binding/unbinding process, MM/GBSA may be a feasible choice.

Human protein kinases regulate a variety of essential physiological processes, including proliferation, invasion, angiogenesis and metastasis, etc^1^,^2^,^3^,^4^, making them important targets for drug discovery. All protein kinases share a structurally conserved catalytic domain which is composed of two major sub-domains, namely the N-terminal and the C-terminal lobes^5^,^6^. The two lobes are connected through a flexible linker region (or hinge region). The activation loop belonging to the C-terminal lobe and adjacent to the linker region regulates the conformational transition between the “on” state (active conformation) and the “off” state (inactive conformation) of the kinases. The ATP-binding site is located in the cleft between the two lobes and the linker region. Most small molecule inhibitors of kinases are known as type I inhibitors which target the ATP-binding pocket in the active conformation. In recently years, the crystal structures of imatinib^7^, sorafenib^8^, and BIRB796^9^ have revealed another kind of kinase inhibitors that occupy both the ATP-binding pocket and the adjacent hydrophobic pocket (also called allosteric pocket) and thereafter were named as type II inhibitors^10^. The type II inhibitors can prevent the kinase activation by binding to the inactive conformations of kinases.

When a type I inhibitor occupies the ATP-binding pocket, the activation loop adopts the conformation that exposes the ATP-binding pocket completely. Then the entry/exit pathway of the type I inhibitor in the active kinase is defined as the ATP-pocket channel. Whereas, when a type II inhibitor targets an inactive kinase, the conformational transition of the activation loop and the conserved DFG (Asp-Phe-Gly) motif will shrink the ATP cleft and create an allosteric pocket. Thus, the ATP-pocket channel narrows and another entry/exit pathway named as the allosteric-pocket channel appears (Figure 1A). Numerous studies have focused on the ATP pocket for the dissociations of type I inhibitors^11^,^12^,^13^. For instance, Capelli *et al*. have studied two type I inhibitors unbinding from the ATP channel from VEGFR2 by using steered molecule dynamics (SMD) simulations^12^, and we have exploited the free energy profile change of a type I inhibitor targeting ALK tyrosine kinase against a series of drug resistant mutations along the ATP pathway by using adaptive biasing force (ABF) simulations^13^. However, which one is the real pathway for the type II inhibitors to move into/out of the target is still unclear.

p38 MAP kinase is one of the most investigated kinases targeted by type II inhibitors, and the crystal structures in complex with type II inhibitors are also available (PDB entries: 1KV1 and 1KV2)^9^. Another reason why p38 MAP kinase can be used as a model system is that its allosteric pocket has been frequently studied^14^,^15^,^16^,^17^,^18^. For example, Wang *et al*. have characterized the free energy landscape of the allosteric pocket of p38 MAP kinase transferred from the DFG-in state to the DFG-out state by using normal mode analysis combined with umbrella sampling (NMA-US)^18^. Thereby, in this study, enhanced sampling simulations (umbrella sampling, US) were employed to investigate how two type II inhibitors (**1** and **BIRB796** shown in Figure 1) unbind from the active pocket through the ATP channel and the allosteric channel, and then the Weighted Histogram Analysis Method (WHAM) was used to generate the potential of mean force (PMF) along the unbinding pathways. Meanwhile, the Molecular Mechanics/Generalized Born Surface Area (MM/GBSA) method was also used to calculate the binding free profiles of the studied inhibitors along the unbinding pathways for a comparison.

## Methods

### Conventional Molecular Dynamics (MD) Simulations

The crystal structures of two type II inhibitors (**1** and **BIRB796**) in complex with p38 MAP kinase (PDB entries: 1KV1 and 1KV2^9^) were employed as the initial structures for the conventional MD simulations. The inhibitors were optimized and then the electrostatic potentials were calculated at HF/6-31G* level of theory supported in Gaussian 09. The partial charges were obtained by fitting the electrostatic potentials using the RESP technique^19^.

The AMBER03 force field^20^ was employed for the protein and the general AMBER force field (*gaff*)^21^ was used for the ligands. 7 Na^+^ were added to both the systems to neutralize the redundant net charges. Each system was immersed into a rectangular TIP3P water box^22^ that is extended 12 Å away from any solute atoms. The Particle Mesh Ewald (PME)^23^ algorithm was used to handle the long-range electrostatic interactions, and the SHAKE^24^ algorithm was employed to constrain the covalent bonds involving hydrogen atoms. The time step was set to 2 fs. The cutoff of 10 Å was used for the short range interactions (van der Waals and electrostatic interactions).

Before MD simulations, steepest descent energy minimization (1000 cycles) and conjugate gradient energy minimization (4000 cycles) were carried out to optimize the systems. Then in the phase of MD simulations, each system was gradually heated from 0 to 300 K over the first 50 ps, and followed by another 50 ps equilibrium simulation in the NPT ensemble (*T* = 300 K and *P* = 1 atm). In the two stages of MD simulations, the heavy atoms of the protein backbone were restrained with the elastic constant of 5 kcal/mol·Å^2^. Finally, a 10 ns production run without any constrain was performed in the NPT ensemble (*T* = 300 K and *P* = 1 atm). All the molecular mechanics (MM) minimizations and MD simulations were performed using the *sander* module in AMBER11^25^.

### Umbrella Sampling Simulations

It is well known that the simulated systems are easily trapped in local minima, and the sampling of some conformational transition processes, such as the unbinding process of a ligand, becomes a very hard task for conventional MD simulations. Thereby, it might need even millisecond level of conventional MD simulations to investigate the transition process for a small system^26^,^27^. Fortunately, the enhanced sampling methods, such as US^28^,^29^,^30^,^31^, metadynamics^32^,^33^, and adaptive biasing force (ABF)^34^,^35^, emerge as smart approaches to solve this problem, through adding either biasing potentials or biasing forces at the certain position of the reaction coordinate (RC) to enhance the sampling of the regions involved in high potential barriers. Take US as an example, to fully investigate the RC, the whole RC should be divided into a series of continuous windows. For convenience, harmonic potential, as shown in the equation below, is added to the original potential (unbiased potential) in each window to drive the system from one thermodynamic state to another. 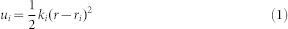
where *u_i_* is the biased potential with respective to the current position *r*. *r_i_* is the reference position in window *i*, and *k_i_* is the elastic constant used to pull the biased molecule out of the binding pocket. Here, an elastic constant of 5 kcal/mol·Å^2^ was used in all the US simulation windows. By adding the biasing potentials, some transition states with high energy barriers can also be fully sampled. To construct the potential of the mean force (PMF) along the RC, WHAM^36^,^37^ was employed to rebuild the biased probability distribution to a normal one. Herein, the RC was separated into 2000 bins for the WHAM calculation after each cycle of the US simulation (a 41 ns US run was defined as a cycle as shown below). The *tolerance* for iteration was set to 0.0001 to get convergent PMF curves. The *temperature* was set to 300 K to keep consistence with the simulation temperature.

**Figure 1:**
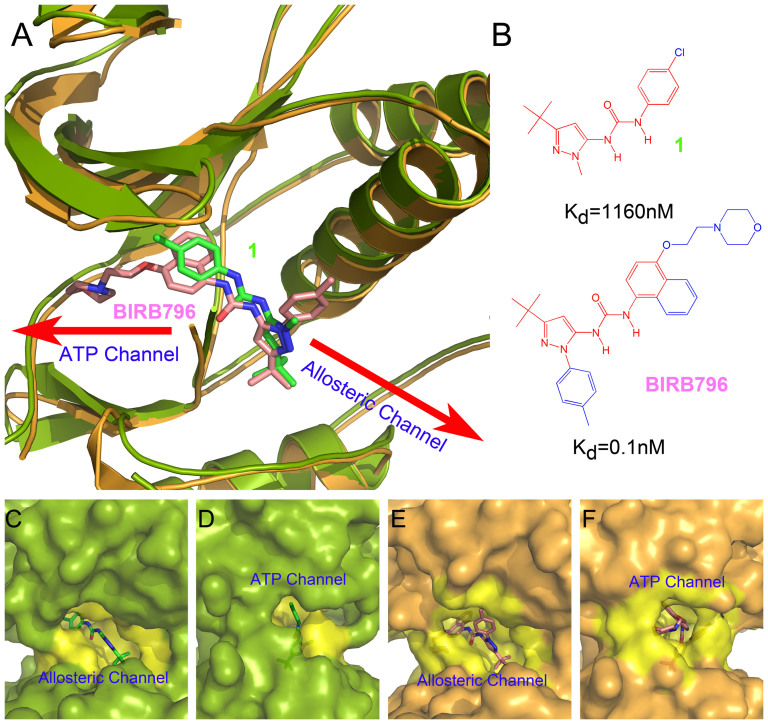
Superimposition of crystallized p38 MAP kinases complexed with ligands 1 (green stick and cartoon models, PDB code: 1KV1) and BIRB796 (pink stick and orange cartoon models, PDB code: 1KV2) (A). The binding affinities of the ligands to p38 MAP kinase are very different as reported by reference [9], where the same part of the two molecules are colored in red (B). Surface models were shown to highlight (yellow region) the allosteric channels (panels C and E for **1** and **BIRB796**, respectively) and ATP channels (panels D and F for **1** and **BIRB796**, respectively).

In this study, two sets of US simulations were carried out to simulate the unbinding processes of two type II inhibitors through the ATP channel and the allosteric channel. During the simulations, the distance between one atom in receptor (O in Ile80 in 1KV1 for the allosteric pathway, C_α_ in Gln304 in 1KV1 for the ATP pathway, C_α_ in Gly81 in 1KV2 for the allosteric pathway, and O in Arg66 in 1KV2 for the ATP pathway) and another atom in ligand (C8 in **1** in 1KV1 for the allosteric pathway, C1 in **1** in 1KV1 for the ATP pathway, N1 in **BIRB796** in 1KV2 for the allosteric pathway, and C4 in **BIRB796** in 1KV2 for the ATP pathway) was selected as the RC, and the vector between the two atoms represents the channel direction (the allosteric channel in Figures 1C and 1E and the ATP channel in Figures 1D and 1F). Each set of the simulations contains 41 simulation windows with 0.5 Å in length for each and the RCs were extended 20 Å away from the initial distances. For each window, 17 ns and 10 ns US simulations were preformed for the systems of **1** (1KV1) and **BIRB796** (1KV2), respectively, to converge the sampling. Thereby, approximate 700 ns (697 ns) and 410 ns US simulations were preformed for systems **1** and **BIRB796**, respectively, which both contain the simulations for the ATP channel and allosteric channel, and a total of >2.2 μs US simulations were performed in this study. To prevent the drifting of the systems, a restraint of 5 kcal/mol·Å^2^ was added to the heavy atoms that are 18 Å away from the ligands in the initial structures.

It has been proposed to use the constrained scheme for the calculations of absolute binding free energy^38^,^39^,^40^, but in this study we did not try to use these methods to calculate the binding free energy due to the fact that the constrained simulations may miss important metastable states when unbinding a ligand from the active site based on the prior determined straight RC^13^. Nevertheless, the purpose of this study is to reveal which pathway is more favorable for the ligand dissociation/entry, and it is true that the difference of the absolute binding free energies is always reflected in the separation degree of freedom^41^. Therefore, the PMF calculated here for the comparison is actually the PMF depth rather than the absolute binding free energy.

### MM/GBSA Binding Free Energy Calculation

To give a comparison with the probability-based PMF estimated from US simulations (estimated by WHAM), two-end-state free energy calculations were preformed directly based on the trajectories derived from the US simulations. Here, the widely used MM/GBSA methodology was employed for the two-end-state free energy calculation^42^,^43^,^44^,^45^,^46^,^47^,^48^,^49^,^50^. The binding free energy (Δ*G*_bind_) between a ligand and a receptor can be calculated according to the following equations^47^. 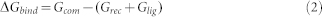

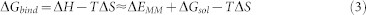

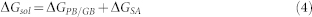
where Δ*E*_MM_ is the gas-phase interaction between protein and ligand, including the electrostatic and the van der Waals energies; Δ*G*_GB_ and Δ*G*_SA_ are the polar and non-polar components of the desolvation free energy, respectively; -*T*Δ*S* is the change of conformational entropy upon ligand binding at temperature *T*, which was not considered here due to the expensive computational cost and low prediction accuracy^44^. Here, the electrostatic solvation energy (Δ*G*_GB_) was calculated by the GB model developed by Onufriev (*igb* = 2)^51^, because this GB model usually outperforms the other GB models used in AMBER^44^. The exterior dielectric constant was set to 80 and the solute dielectric constant was set to 1. The non-polar contribution of the desolvation energy (Δ*G*_SA_) was estimated from the solvent accessible surface area (SASA) using the LCPO algorithm: Δ*G*_SA_ = 0.0072 × ΔSASA + 0.00^52^.

## Results and discussion

### Convergence of the simulated systems

In order to equilibrate the systems, conventional MD simulations were performed for the complexes of p38 MAP kinase before the US simulations. As shown in Figure S1 in the supporting information, the low root mean square deviations (RMSDs, <1.5 Å on average) of the backbone heavy atoms with respect to the crystal structures suggest that the studied systems are stable throughout the 10 ns conventional MD simulations, and thereby suitable for the following US analyses. The last snapshots of the conventional MD trajectories were chosen as the initial conformations for the US simulations.

**Figure 2:**
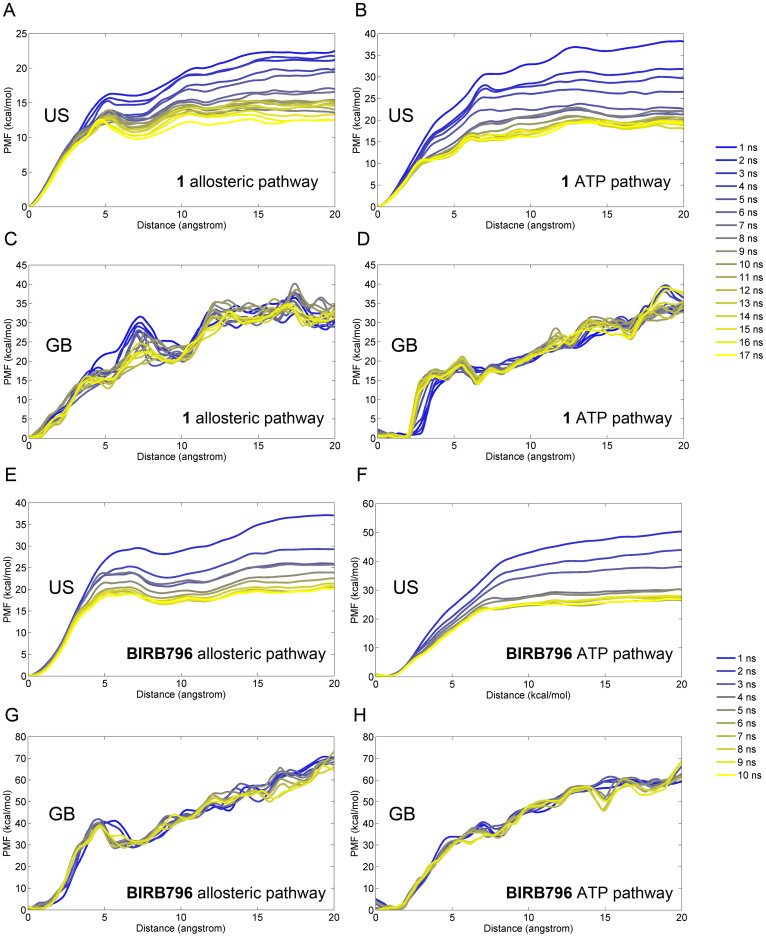
Convergence of the PMFs calculated by umbrella sampling (panels A, B, E, and F) and MM/GBSA (panels C, D, G, and H), where 17 ns and 10 ns US simulations were performed for the complex of 1 and BIRB796, respectively.

To guarantee the sampling convergence of the US simulations, 17 ns and 10 ns US simulations for each window were preformed for the systems of **1** and **BIRB796**, respectively, and the convergence of PMF was checked after each nanosecond of simulations. As shown in Figure 2, 17 and 10 curves were plotted for the systems of **1** (Figures 2A~2D) and **BIRB796** (Figures 2E~2H), respectively. It can be found that the system of **1** converged after approximate 10 ns of the US simulations, and 5 ns of the system **BIRB796**, with the PMF difference less than 1 kcal/mol (the PMF values were estimated based on 15 ~ 20 Å of the RCs). Interestingly, the PMF curves estimated by MM/GBSA showed convergence at the beginning of calculation. As shown in Figures 2C~2F, all the curves overlap in the same region and similar behaviors were found between the US and the MM/GBSA based PMF curves. For instance, when **BIRB796** dissociates from the allosteric pathway, a low energy barrier was found in both the US results (Figure 2E) and the MM/GBSA results (Figure 2G) at ~5 Å of the RC, suggesting that MM/GBSA with much less computational cost is a feasible approach to calculate the free energies along a RC^53^. Nevertheless, due to the natural characteristics of the algorithm, the free energies calculated by MM/GBSA are much larger than those calculated by US (WHAM) and the experimental data (Table 1), most of which may be attributed to the ignorance of the contributions of the conformational entropies to the binding free energies^54^.

### Binding free energies along allosteric and ATP channels

**Figure 3:**
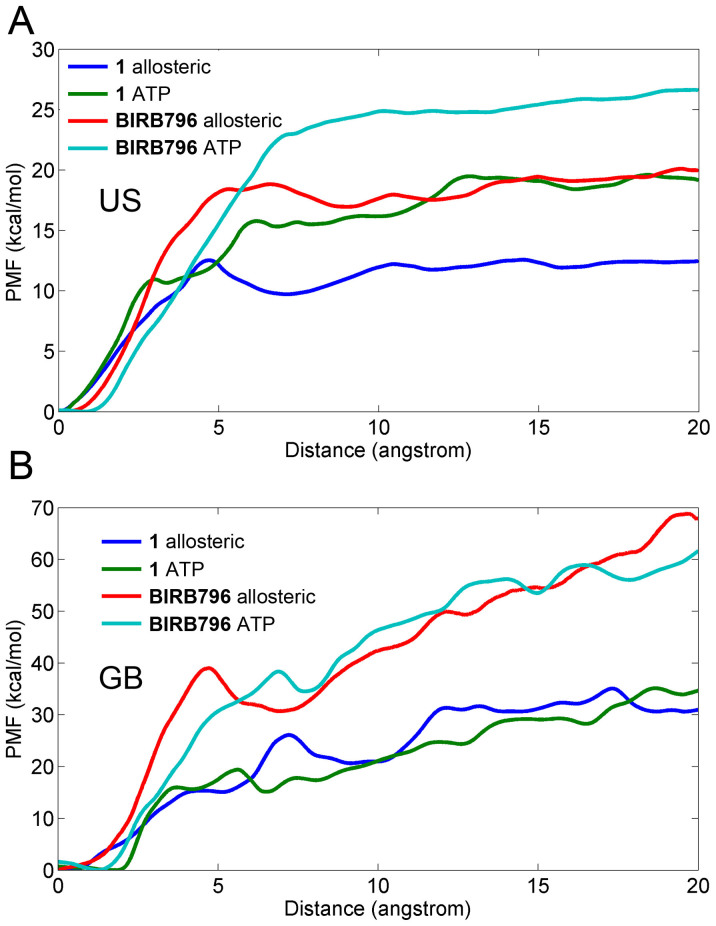
Comparison of the PMFs based on different dissociation pathways using US simulations (A) and MM/GBSA calculations (B). The last 1 ns simulation results were used for the comparison.

As shown in Figure 1, the same core structures (red fragments in panel B) and binding poses (panel A) were employed by the two inhibitors (**1** and **BIRB796**). However, the extended fragments of **BIRB796** (blue fragments) greatly enhances its binding affinity (from 1160 nM to 0.1 nM) to p38 MAP kinase as reported by Pargellis *et al*.^9^. As shown in Table 1 and Figure 3, the ranks of the binding free energies along both the allosteric pathway and the ATP pathway predicted by US (WHAM) and MM/GBSA are consistent with that of the experimental data. Compared with the US results, the free energies predicted by MM/GBSA along the two directions (allosteric and ATP pathways) are more consistent, with the difference of the binding free energies less than the standard deviations, but are much larger and unable to reach the flat phase of the PMF after the inhibitors dissociated from the target, especially the PMF curves of **BIRB796** (Figure 3B cyan and red curves), which increase continuously after the molecule getting out of the binding pocket (Figures 5E and E′). As mentioned above, this phenomenon may be explained by the intrinsic limitations of MM/GBSA, which employs a parameterized implicit solvent model and uses some arbitrary parameters (post-processing parameters) to calculate the free energy, such as using different salt concentration and dielectric constants (such as *ε*_solute_ = 1 and *ε*_solvent_ = 80)^54^,^55^, implying that there is still interaction even the ligand moves far away from the receptor (20 Å). However, US is a probability based method, and the probability distribution can be derived directly from the simulation trajectory which depends only on the original simulation settings (without using another post-processing method), for example, using the explicit solvent model (TIP3P water) which is closer to a real system. Therefore, the PMF curves derived from US should be closer to the reality and the experimental data (Table 1).

As shown in Table 1 and Figure 3A, the PMF values derived from the US simulations along the two channels are quite different for the two inhibitors (with the energy difference more than 6 kcal/mol and much larger than the standard deviations). Therefore, the allosteric channel is apparently more favorable for the dissociations of the two inhibitors. Interesting, along either pathway, the binding affinities of the two inhibitors can be correctly ranked, such as −19.03 kcal/mol for **1** *versus* −26.06 kcal/mol for **BIRB796** along the ATP channel, and −12.34 kcal/mol for **1** *versus* −19.57 kcal/mol for **BIRB796** along the allosteric channel. That is to say, correct ranking for different molecules may be always resulted once a same dissociation pathway was employed, though the binding free energies along different pathways may be quite different. Nevertheless, the binding free energy difference (ΔPMF_US_) between the two molecules (**1** and **BIRB796**), which is 7.03 or 7.23 kcal/mol along the ATP or allosteric pathway, (Table S1) is in good consistence with the experimental binding affinity difference of 5.56 kcal/mol (ΔΔ*G*_exp_), well supporting the opinion that same dissociation pathway should be employed when determining the relative binding affinities of a set of ligands.

### Dissociation processes of *1* and *BIRB796* along the allosteric and ATP channels

**Figure 4:**
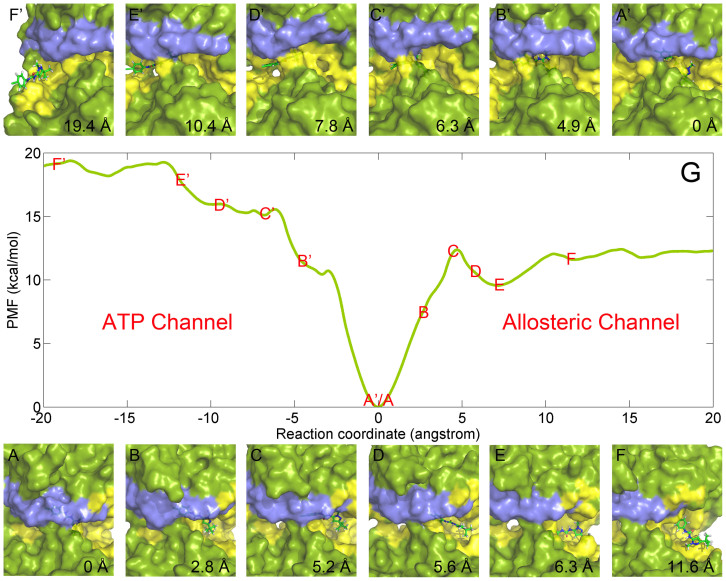
Unbinding process of 1 getting out of the binding site of p38 MAP kinase (panel G) through allosteric channel (panels A~F) and ATP channel (panels A′~F′), where the binding site and the P-loop region are colored in yellow and blue, respectively.

**Figure 5:**
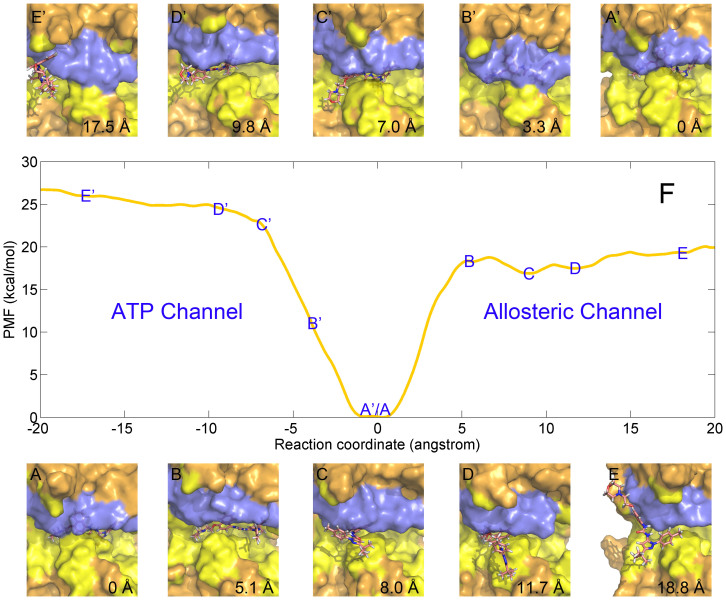
Unbinding process of BIRB796 getting out of the binding site of p38 MAP kinase (panel F) through allosteric channel (panels A~E) and ATP channel (panels A′~E′), where the binding site and the P-loop region are colored in yellow and blue, respectively.

To determine the favorable pathway for the two inhibitors (**1** and **BIRB796**) unbinding from/binding to the target, PMFs derived from the last 1 ns US simulations were connected between the allosteric pathway and the ATP pathway as shown in Figures 4G and 5F. The lowest value of the PMFs (0 kcal/mol) were calibrated to 0 Å on the RCs to denote the bound states of the inhibitors. The RCs were extended from 0 to −20 Å and 0 to 20 Å to represent the inhibitors 20 Å away from the ATP channel side and the allosteric channel side of the binding pocket, respectively. In theory, the PMF values should be equal everywhere once the ligands move far away from the binding site (20 Å away from the binding pocket) due to the isotropic bulk. However, as shown in Figures 4G and 5F, the PMF values along the ATP pathway are actually much higher than those along the allosteric pathway for the two ligands (both more than 6 kcal/mol as shown in Table 1), indicating that it may be more favorable for the small molecules dissociating along the allosteric pathway due to the much lower PMF depth (0 ~ 20 Å of the RC in Figures 4G and 5F) and more similar binding free energies to the experimental data (Table 1). As shown in Figures 1C~1F, the allosteric pocket (panels C and E) is obviously larger than the ATP pocket (panels D and F), explaining why much lower PMFs were produced when the inhibitors dissociated from the allosteric pathway and supporting the general opinion that the largest pocket pathway is usually considered as the binding/unbinding pathway^13^,^41^,^56^. The reason why the PMFs along the ATP pathway are much larger than those along the allosteric pathway may be partially related to the hard sampling of some associated metastable states of the simulations. As discussed by St-Pierre *et al.*, it will be hard to generate reliable PMF curves when selecting an unfavorable pathway even the convergence of sampling is achieved^57^. Nevertheless, the hard sampling of certain pathways may be an efficient way to find the favorable pathway for the ligand entry/dissociation. However, as mentioned above, the PMFs derived from MM/GBSA are more consistent, and thereby making it hard to determine which way is more favorable for the binding/unbinding of the inhibitors.

As shown in Figures 4G and 5F, different unbinding processes were observed for the two molecules though similar core structures were employed by them (red fragments in Figure 1B). When **1** escapes through the allosteric pathway, an obvious energy barrier is shown at point C of the PMF profile (5.2 Å of the RC in Figure 4G), where the methyl head (**1** in Figure 1B) gradually gets out of the binding pocket (Figures 4B and 4C) to the more widely metastable outside binding site (6.3 Å of the RC in Figure 4E). As the large part of the molecule moves out of the binding pocket (Figure 4D), the ligand rapidly absorbs on the outside binding position (Figure 4E) with the PMF curve decreasing markedly (point E in Figure 4G). After unbinding from the outside binding position, **1** totally dissociates from the target (Figure 4F). Alternatively, no energy barrier is observed when **1** dissociates from the ATP pathway (0 ~ −20 Å of the RC in Figure 4G). It may be very unfavorable for the ligand unbinding through the ATP pathway because the binding channel is much longer (~13 Å of the ATP channel *versus* ~5 Å of the allosteric channel, Figure 4G) and narrower (Figures 1C and 1D) compared with the allosteric pathway. As shown in Figure 4G, three stages of steep upgrading of the PMF (regions of A′~B′, B′~C′, and D′~E′ of the RC) are observed before **1** dissociating from the ATP channel of the target, where the conformation of the ligand is pulled from the curved state to flat state through the three stages of dissociation (Figures 4A′~4F′).

Compared with the PMF profile of **1**, the PMF curve of **BIRB796** is much smoother with only a low energy barrier at approximate 5 Å of the RC (Figure 5B) when the ligand unbinds from the allosteric pathway, where the conformation of the ligand is pulled to be nearly flat which corresponds to a high strain energy of the molecule. However, a rotation phenomenon is observed at the next time step probably because of the high conformational energy of **BIRB796**. The ligand opens the P-loop region of the kinase (blue region in Figure 5C) to relax the high strain energy of the flatted ligand which corresponds to a local minimum of the system as shown in C point of the PMF curve in Figure 5F. After that, the molecule dissociates vertically to the P-loop region to the bulk (Figures 5C and 5D). As for the ATP channel, a continued rising PMF without any plain state is observed when the molecule dissociates from the ATP channel (Figure 5F). Although the rising rate of the PMF (slope) seems to be moderated (point C′ of Figure 5F) when the N and O containing heterocyclic tail (Figure 1B) gets out of the ATP channel (Figure 5C′), the PMF curve rises continuously till the bifurcated head, which contains a benzene and a methyl with the angle >120° connected to the double N hetero-ring (pink stick model in Figure 1A), leaves the narrow channel (after the D′ point of the PMF profile as shown in Figure 5F). Thereby, the large bifurcated head of BIRB796 is not favorable to dissociate from the ATP channel, though similar unbinding lengths of the allosteric (> 8 Å) and the ATP (~10 Å) channels are observed in the PMF profile in Figure 5F.

## Conclusion

By using enhanced sampling simulations and two-end-state calculations, we systemically investigated the unbinding processes of two type II inhibitors escaping from p38 MAP kinase through two pathways, namely the ATP pathway and the allosteric pathway. Consistent with the general idea that the largest channel is usually the probably entry/exit pathway for a ligand binding/dissociation, we found that the PMF depths of the allosteric channel for the two inhibitors are both much lower than those of the ATP pathway, indicating that the inhibitors may dissociate/bind through the allosteric channel. Besides, although the ATP channel may be not a favorable pathway for the ligands dissociation, right ranking of the binding free energies of the two distinct inhibitors was also observed (PMFs based on US), implying that similar (binding/unbinding) pathway should be used for the binding affinity comparison.

Moreover, although MM/GBSA may result in much larger binding free energies compared with the experimental data, it is much easier to gain convergent PMFs compared with the much time consuming US simulations and keeps similar shape between the PMF profiles based on US as well. Thereby, to detect important transition state, MM/GBSA may also be a feasible choice to calculate the binding free energies along a RC.

## Author Contributions

Conceived and designed the experiments: T.H. Performed the experiments: H.S., S.T., S.Z., Y.L., D.L., L.X., M.S. and P.P. Analyzed the data: H.S., S.T., S.Z., Y.L., D.L., L.X., M.S. and P.P. Contributed reagents/materials/analysis tools: H.S., S.T., S.Z., Y.L., D.L., L.X., M.S. and P.P. Wrote the paper: H.S. and T.H.

## Supplementary Material

Supplementary Information Suppporting Materials

## Figures and Tables

**Table 1:** PMFs of the ligands unbinding from the ATP channel and the allosteric channel based on the US simulations and MM/GBSA calculations (kcal/mol)

Name	Pathway	PMF_US_^a^	ΔΔ*G*_US_^c^	Δ*G*_GB_^b^	ΔΔ*G*_GB_^c^	Δ*G*_exp_
1	ATP Pathway	−19.03 ± 0.39	6.69	−31.70 ± 2.50	−0.41	−8.11 (1160 nM)
	Allosteric Pathway	−12.34 ± 0.18		−32.11 ± 1.35		
BIRB796	ATP Pathway	−26.06 ± 0.37	6.49	−57.65 ± 1.66	−3.64	−13.67 (0.1 nM)
	Allosteric Pathway	−19.57 ± 0.33		−61.29 ± 4.68		

^a^The PMF_US_s and the standard deviations were estimated by averaging the ensemble energy from 15 to 20 Å reaction coordinate based on the last 1 ns US simulations.

^b^The PMF_GB_s and the standard deviations were calculated by using MM/GBSA approach based on the 15 ~ 20 Å reaction coordinate of the US trajectories.

^c^The energy differences were calculated along the two channels by ΔΔ*G*_(US/GB)_ *= * PMF_allosteric_ − PMF_ATP_.
