# Supplementary material for: Revealing the favorable dissociation pathway of type II kinase inhibitors via enhanced sampling simulations and two-end-state calculations
Source: Sci Rep. 2015 Feb 13;5:8457. doi: 10.1038/srep08457 (PMC4326958; doi:10.1038/srep08457)
Supplement: Supplementary Information — Suppporting Materials [file srep08457-s1.doc]

**Revealing the favorable dissociation pathway of type II kinase inhibitors *via* enhanced sampling simulations and two-end-state calculations**

Huiyong Sun*^a,b,#^*, Sheng Tian*^a,#^*, Shunye Zhou*^a^*, Youyong Li*^a^*, Dan Li*^b^*, Lei Xu*^a^*, Mingyun Shen*^a^*, Peichen Pan*^a^* and Tingjun Hou*^a,b^,**

^a^Institute of Functional Nano and Soft Materials (FUNSOM), Soochow University, Suzhou, Jiangsu 215123, P. R. China. ^b^College of Pharmaceutical Sciences, Zhejiang University, Hangzhou, Zhejiang 310058, P. R. China.

**Supporting Information**


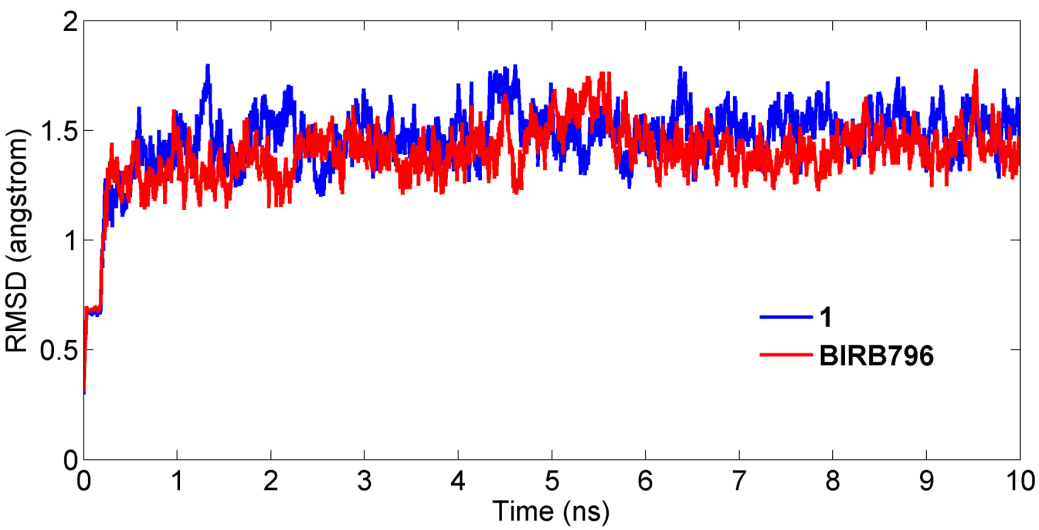


**Figure S1.** Root mean square deviations (RMSD) of heavy atoms in backbone of p38 MAP kinase along 10 ns conventional MD simulations. The systems of **1** and **BIRB796** were colored in blue and red, respectively.

**Table S1.** Energy difference between experimental data and PMF values based on US (kcal/mol).

| **Name** | **Pathway** | **PMF_US_^a^** | **ΔPMF_US_^b^** | **Δ*G*_exp_** | **ΔΔ*G*_exp_^c^** |
| --- | --- | --- | --- | --- | --- |
| **1** | ATP Pathway | -19.03±0.39 | 7.03 | -8.11 | 5.56 |
| **BIRB796** |  | -26.06±0.37 |  | -13.67 |  |
| **1** | Allosteric Pathway | -12.34±0.18 | 7.23 | -8.11 | 5.56 |
| **BIRB796** |  | -19.57±0.33 |  | -13.67 |  |

^a^The PMF_US_s and the standard deviations were estimated by averaging the ensemble energy from 15 to 20 Å reaction coordinate based on the last 1 ns US simulations.

^b^The expected binding free energy differences were calculated by ΔPMF_US_ *=* PMF**_1_** – PMF**_BIRB796_**.

^c^The experimental binding free energy difference was calculated by ΔΔ*G*_exp_ *=* Δ*G***_1_** – Δ*G***_BIRB796_**.
